# Generation of ultra-large-bandwidth X-ray free-electron-laser pulses with a transverse-gradient undulator

**DOI:** 10.1107/S1600577516007177

**Published:** 2016-05-13

**Authors:** Eduard Prat, Marco Calvi, Sven Reiche

**Affiliations:** a Paul Scherrer Institut, CH-5232 Villigen PSI, Switzerland

**Keywords:** free-electron laser, ultra-large-bandwith X-ray pulses, transverse-gradient undulator, simulations

## Abstract

A new and simple method to generate X-ray free-electron-laser radiation with unprecedented spectral bandwidth above the 10% level is presented. The broad bandwidth is achieved by sending a transversely tilted beam through a transverse-gradient undulator. The extent of the bandwidth can easily be controlled by variation of the beam tilt or the undulator gradient. Numerical simulations confirm the validity and feasibility of this method.

## Abstract 2

A novel, flexible yet simple method to generate gigawatt X-ray free-electron-laser radiation with unprecedented spectral bandwidth above the 10% level is presented. Such broadband radiation will improve substantially the efficiency of techniques like X-ray crystallography and spectroscopy, paving the way for outstanding progress in fields like biology and material science.

## 1. Introduction   

X-ray free-electron-laser (XFEL) facilities are leading-edge scientific instruments in multiple research fields like biology, material science, chemistry and physics. XFELs are commonly based on the self-amplified spontaneous emission (SASE) process (Kondratenko & Saldin, 1980 ▸; Bonifacio *et al.*, 1984 ▸). SASE-XFELs produce transversely coherent radiation with pulse powers of tens of gigawatts and pulse durations of a few tens of femtoseconds and shorter (Emma *et al.*, 2010 ▸). The natural bandwidth of the SASE-XFEL pulses is of the order of the Pierce parameter ρ (Bonifacio *et al.*, 1984 ▸), with values between 10^−3^ and 10^−4^ for a typical XFEL facility. To improve the longitudinal coherence of SASE-XFELs, seeding techniques are employed, which further reduce the radiation bandwidth close to the Fourier limit (Feldhaus *et al.*, 1997 ▸; Saldin *et al.*, 2001 ▸; Geloni & Saldin, 2010 ▸; Amann *et al.*, 2012 ▸).

There is, however, a strong scientific interest in obtaining large-bandwidth XFEL pulses for some applications such as X-ray crystallography (Dejoie *et al.*, 2013 ▸; Baradaran *et al.*, 2012 ▸; Arthur *et al.*, 2013 ▸), X-ray emission and absorption spectroscopy (Patterson *et al.*, 2010 ▸), stimulated Raman spectroscopy (Baradaran *et al.*, 2012 ▸) and multi-wavelength anomalous diffraction (Son *et al.*, 2011 ▸). The generation of broadband XFEL radiation will improve significantly the efficiency of these techniques, paving the way for outstanding progress in research fields such as biology and material science. From the operation point of view, a broad bandwidth allows for more flexibility in the use of the radiation: the XFEL wavelength can be tuned by monochromators without the need to change any parameter on the accelerator side.

The central wavelength λ of the XFEL radiation is given by the resonance condition (Bonifacio *et al.*, 1984 ▸),
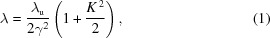
where 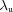 is the undulator period length, γ is the Lorentz factor of the electron beam and *K* is the undulator field parameter. A natural way to obtain large-bandwidth XFEL pulses is to produce the radiation with an energy-chirped electron beam; *i.e.* when in the beam there is a correlation between the energy and the longitudinal position of the electrons. For instance, according to equation (1), a 1% electron energy chirp will generate XFEL pulses with 2% wavelength bandwidth. Andonian and co-workers obtained radiation pulses at wavelengths in the micrometer range with a total bandwidth of up to 15% using an electron beam with a central energy of 70 MeV and a large energy chirp (Andonian *et al.*, 2005 ▸). In the X-ray regime the electron beam energy needs to be much higher, of the order of gigaelectronvolts. Consequently, obtaining a large energy chirp is much more difficult. Different approaches exist for obtaining the energy chirp at XFEL facilities: optimizing the compression scheme of the linac and using the longitudinal wakefields of the RF structures, as considered in various facilities such as SwissFEL (Saá Hernández *et al.*, 2016 ▸) or the Linac Coherent Light Source (Turner *et al.*, 2015 ▸); modifying the longitudinal laser profile of the photoinjector (Penco *et al.*, 2014 ▸; Saá Herñandez *et al.*, 2016 ▸); and utilizing the collective effects of an extremely compressed beam, as proposed for the European XFEL project by Serkez *et al.* (2013 ▸). By using these methods, or combining them, the bandwidth of the XFEL pulses can reach the few-percent level. A possibility to further increase the energy chirp of the beam is given by exploiting the wakefield of dedicated beamline elements such as corrugated pipes and dielectric structures (Craievich, 2010 ▸; Emma *et al.*, 2014 ▸; Antipov *et al.*, 2014 ▸; Zhang *et al.*, 2015 ▸). For practical reasons the energy chirp of the electron beam can only be increased up to a few percent, otherwise dispersive and chromatic effects will prevent the amplification of the XFEL process.

## 2. Description of the scheme   

**Figure 1:**
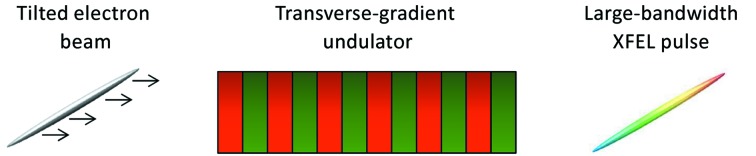
Sketch to show the working principle of the proposed method: an electron beam with a transverse tilt traveling through a TGU generates large-bandwidth XFEL radiation.

We propose a more efficient way to produce large-bandwidth XFEL pulses by using a transverse-gradient undulator (TGU) (Smith *et al.*, 1979 ▸; Fuchert *et al.*, 2012 ▸) in conjunction with a transversely tilted electron beam. In a TGU there is a linear dependence of the undulator field on the transverse position *x*,
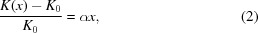
where 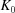 is the on-axis value of the undulator field parameter and α is a constant gradient. In a transversely tilted beam there is a correlation between the transverse and longitudinal positions (time) of the particles. Therefore, if such a beam travels through a TGU it will produce XFEL radiation with broad bandwidth, as shown schematically in Fig. 1 ▸. The transverse tilt can be characterized with the tilt amplitudes in offset d*x*/d*s* and angle d*x*′/d*s*, with *x* being the transverse offset of the electrons, *x*′ their transverse angle, and *s* their longitudinal coordinate.

In our method it is straightforward to tune the bandwidth of the XFEL radiation by modifying the tilt amplitude or the gradient of the TGU or both. This is an additional advantage with respect to previous approaches, which are difficult to set up and to tune (see below for more details).

Increasing the beam size horizontally would also generate broadband radiation with a TGU. However, the increase of the beam size would deteriorate the XFEL performance such that the radiation power would not grow significantly. A transverse tilt overcomes this limitation: with a transverse tilt, although the total beam size is also increased, the transverse size of the volume occupied by the electrons within the cooperation length of the XFEL process (a small fraction of the total bunch length) is almost preserved, therefore the XFEL performance is practically not affected.

In a typical undulator beamline the electrons are focused with quadrupole magnets to have small beam size for an optimum XFEL performance, *i.e.* maximum XFEL power at a minimum undulator length to achieve saturation. If the beam is transversely tilted, focusing would cause only a small fraction of the beam to produce XFEL radiation, *i.e.* the part whose betatron oscillations are small enough to allow a good transverse overlap between the electron and photon beams. This concept has been proposed to reduce the XFEL pulse duration (Emma & Huang, 2004 ▸) and to generate high-power and short XFEL pulses (Prat *et al.*, 2015 ▸). In our case, however, all the electrons need to produce XFEL radiation and the transverse positions of the electrons must be as constant as possible in order for a longitudinal slice to radiate at the same frequency. Therefore all electrons comprising the beam should travel parallel along the undulator axis. This is achieved if the beam has a transverse tilt only in offset (not in angle) at the undulator entrance and in the absence of external focusing all along the undulator lattice. As a result, the betatron functions within the undulator beamline will be larger than the optimum ones. The electron beam will still see the natural focusing of the undulator. If the betatron phase-advance along the undulator beamline is much less than 90°, which is normally the case for electron beams with GeV energies, the change of transverse trajectory due to the undulator focusing can be compensated by tapering the TGU gradient.

In the following we will discuss the implementation of our method and present simulations that demonstrate the feasibility of our scheme. For that we will take as an example the soft X-ray beamline of SwissFEL (Ganter, 2012 ▸), which is expected to produce by the year 2020 XFEL radiation in the wavelength range between 0.7 and 7 nm (photon energies between 0.18 and 1.77 keV). We note, however, that the method works in general for any photon energy. The undulator line consists of Apple-III devices (Clarke, 2004 ▸; Schmidt *et al.*, 2015 ▸) with an undulator period of 40 mm. From the wavelength range we select the case of 1 nm (1.24 keV), which corresponds to a beam energy of 3 GeV and a nominal 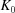 of 1.2.

### 2.1. Generation of the TGU gradient   

**Figure 2:**
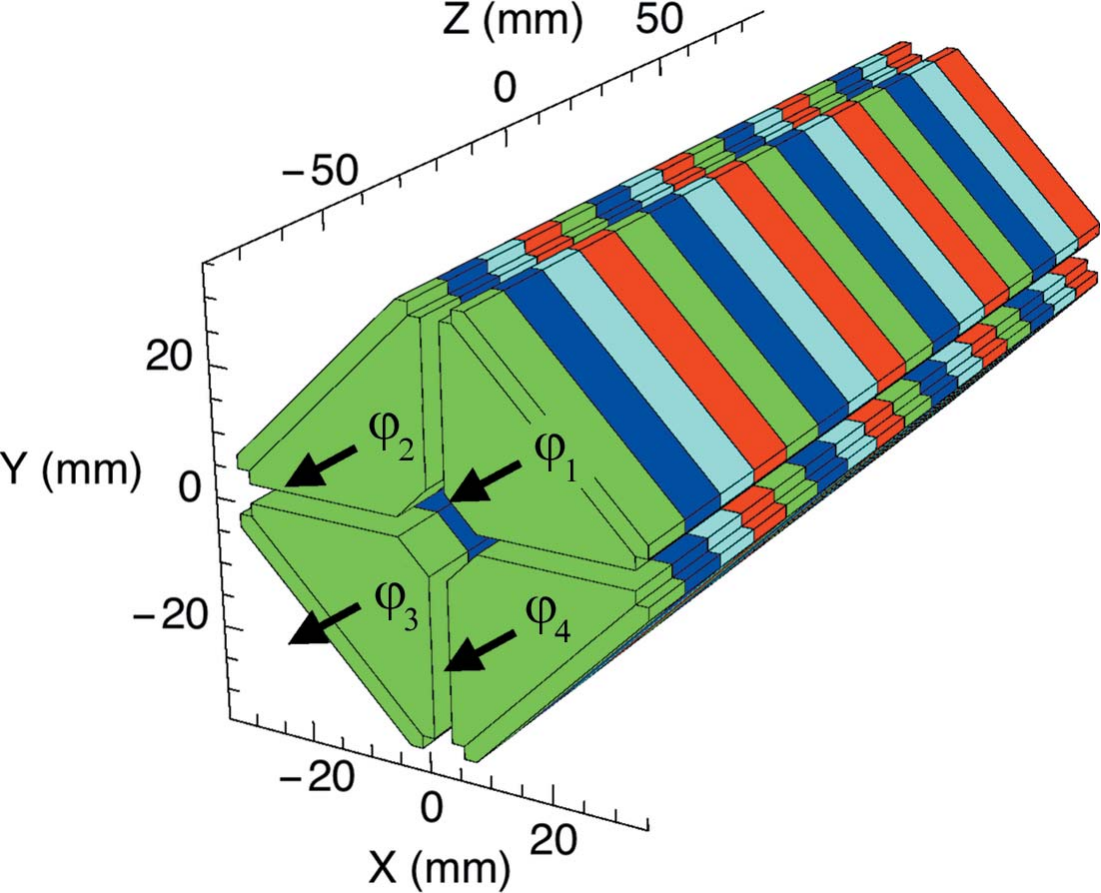
Sketch of the standard Apple undulator device. Each color corresponds to a different magnetization direction and the arrows represent possible longitudinal shifts (with respect to *Z*). See text for more details.

The gradient can be generated with an undulator with canted poles as proposed by Huang *et al.* (2012 ▸), where a gradient of around 40 m^−1^ for a standard undulator and of 150 m^−1^ for a superconducting undulator were considered. A more versatile option consists of employing standard Apple undulators equipped with a variable-gap drive system and four mechanically independent magnetic arrays, as shown in Fig. 2 ▸. The gradient can be obtained by shifting longitudinally either the two upper arrays 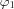 and 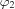 against the lower arrays 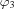 and 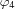 (top–bottom shift), or the two left arrays 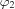 and 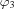 against the two right arrays 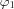 and 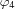 (left–right shift). A top–bottom shift generates a gradient in the horizontal direction while a left–right shift produces a gradient in the vertical plane. The sign of the gradient can be inverted by shifting in the opposite direction. With this method the gradient can easily be controlled by varying the longitudinal shifts. Since 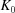 and α are strongly correlated in the case of a fixed-gap device, an undulator with variable gap is required to have independent control over both variables.

The gradient may be determined by measuring the central wavelength of the XFEL radiation with a spectrometer as a function of the transverse offset of the electron beam in the undulator.

**Figure 3:**
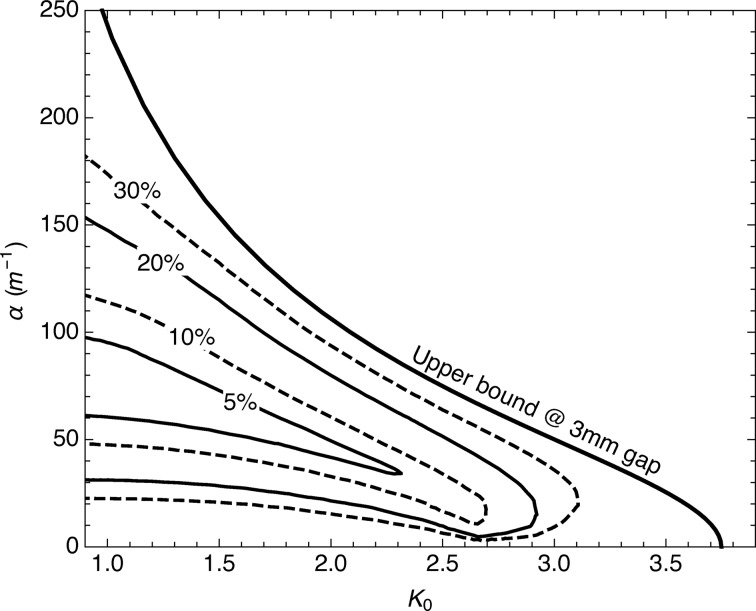
Available gradient for a standard Apple undulator as a function of the undulator field parameter on-axis. The gradient is obtained by shifting longitudinally the arrays of the Apple device. The different lines define areas with a certain level of field linearity, defined as the maximum deviation of the gradient in a region of ±1 mm with respect to the on-axis gradient. For instance, for 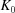 = 1, we can obtain a gradient between 30 and 150 m^−1^ with a linearity better than 20% (see text for more details).

In Fig. 3 ▸ the operational range of 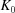 and α for the SwissFEL undulators is presented, as computed using the *RADIA* software package (Chubar *et al.*, 1998 ▸). The simulations are calculated assuming samarium–cobalt magnets with a remanence of 1.08 T. The calculations have been performed for circularly polarized light; for linear polarization the obtained gradients are much smaller. The upper bound is calculated with the undulator at its minimum gap (3 mm) and for top–bottom shifts between 0 and 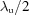 (0 and 20 mm). The transverse area where the gradient is constant, *i.e.* where the field is linear, depends on the working point. In Fig. 3 ▸ the region of ±1 mm has been identified and the deviation from constant gradient is estimated as the maximum change of the gradient with respect to its central value. The results are expressed as percentages. As the figure shows, a gradient between ∼30 m^−1^ and ∼80 m^−1^ with a variation smaller than 20% can be achieved for 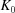 between 1 and 2.

A TGU with a constant gradient will produce a transverse kick to the beam, which can be easily compensated with a dipole field corrector, and a weak natural focusing (Jha & Wurtele, 1993 ▸). For the gradient considered in our study this focusing strength is 20 times weaker than the natural focusing of a standard undulator, therefore we neglect it in our calculations. For a non-constant gradient this weak natural focusing can be increased significantly. For the simulations presented in this paper, however, the field linearity is good enough to neglect any additional focusing effects.

There are two main limitations in using the standard Apple device. The first concerns the polarization, because this technique works only for circularly polarized light. The second concerns the small range of field linearity when operating with low gradients (below 20 m^−1^). Both these problems can be overcome with the development of a new generation of Apple undulators where a split gap system can independently drive the left and the right magnetic arrays. At SwissFEL the further option of four independent gap drive systems is presently under investigation.

### 2.2. Generation of the transverse tilt   

The transverse tilt of the beam can be generated with different methods, all relying on standard components of XFEL facilities: applying a transverse deflecting RF structure to streak the beam (Loew & Altenmueller, 1965 ▸), introducing dispersion to an energy chirped beam (Prat & Aiba, 2014 ▸), or using the transverse wakefields (Zotter & Kheifets, 1998 ▸) of the accelerating or any other structures of the beamline. The beam tilt can be easily tuned if it is generated with a transverse deflector by simply varying the deflector voltage. If the tilt is created with dispersion, one can modify the dispersion strength and compensate the change in optics and/or trajectory accordingly.

The tilt amplitude can be measured by streaking the beam in the plane perpendicular to the tilt plane; for instance, a horizontal tilt would be measured by streaking the beam in the vertical plane. This diagnostic streaking can be achieved with the same methods as used for the tilt generation.

The transverse deflecting structures foreseen for SwissFEL operate at a C-band frequency (*f* = 5.72 GHz) and provide a total integrated voltage of 70 MV (Craievich *et al.*, 2013 ▸). These structures can apply a kick of about ±28 µrad to a 3 GeV electron beam with a total length of 20 µm (*i.e.* a duration of 67 fs). Considering a β-function at the deflector position of 100 m, this corresponds to a tilt amplitude in offset of 125 at the undulator entrance for our beam optics (*i.e.* a trajectory offset variation of ±1.25 mm along the whole bunch length). Stronger tilts can be obtained with the dispersion method, for instance by using a quadrupole magnet in a bunch compression chicane of the facility, where the beam by default has an energy chirp of the order of 1%, as required for the compression process.

## 3. Simulations   

**Figure 4:**
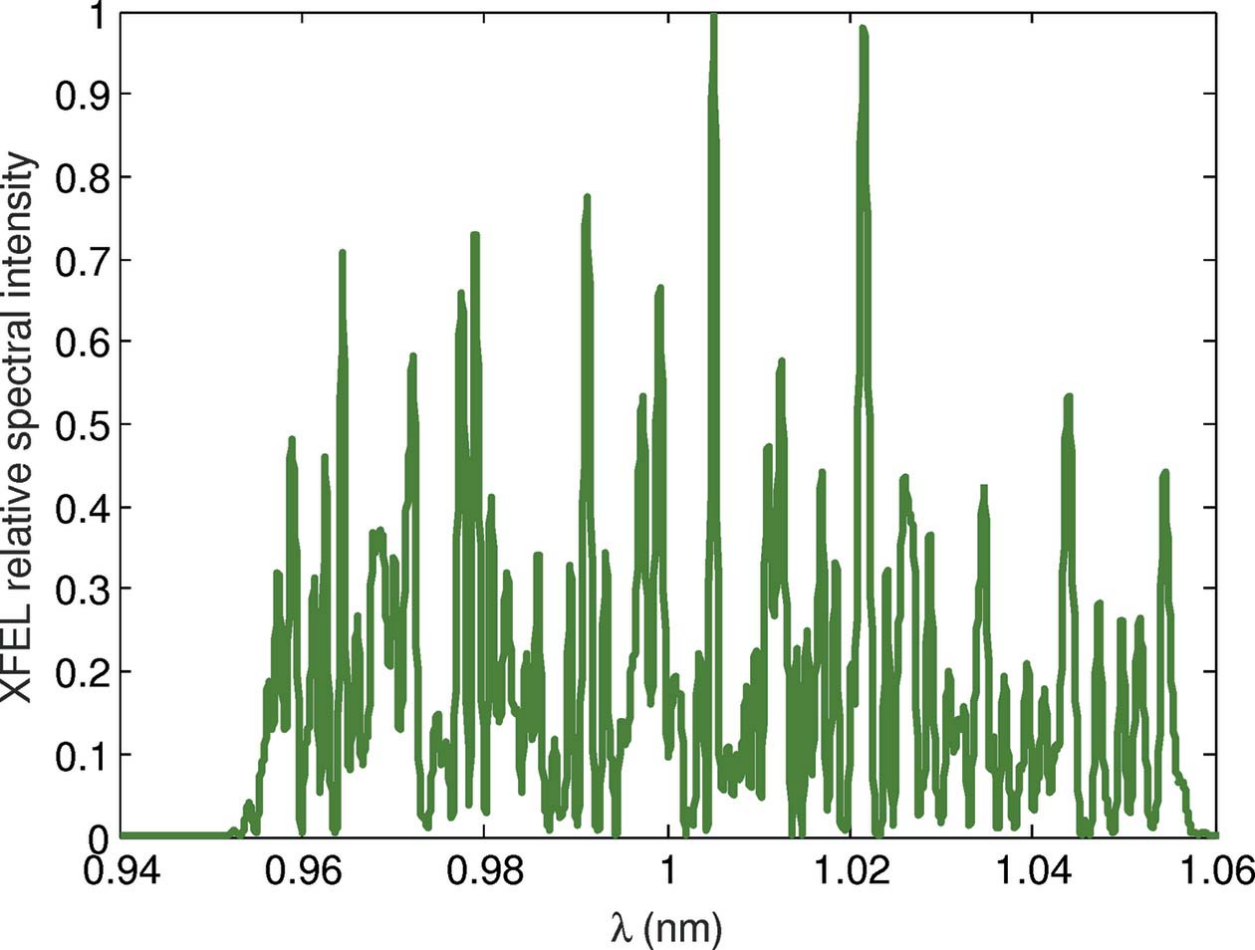
Example of a simulated spectrum for an XFEL pulse with a total bandwidth of 10%. It is obtained with a tilt amplitude in offset of 125 and a TGU gradient of 48 m^−1^.

According to equations (1) and (2), a beam having a tilt amplitude in offset of 125 traveling through a TGU with a gradient of 48 m^−1^ will produce radiation with a total bandwidth of 10%. We have performed numerical simulations using the code *Genesis* 1.3 (Reiche, 1999 ▸, 2015 ▸) to further investigate this case. The simulated electron beam has the following properties: a flat energy profile with a mean value of 3.0 GeV and a slice energy spread of 350 keV, a flat current profile with a value of 3 kA and a total charge of 200 pC (corresponding to a total pulse length of 20 µm or 67 fs), and a normalized transverse emittance of 300 nm. We consider a continuous planar undulator of 40 m length with natural weak focusing in the vertical plane. Equivalent results in terms of bandwidth would be obtained for a helical undulator; the performance in terms of XFEL power, however, would be better for helical undulators thanks to the higher coupling between the electrons and XFEL radiation. For a total undulator length of 40 m without external focusing, the matching β-functions are 40.0 m and 34.7 m in the horizontal and vertical planes, respectively. We have run five simulations using different seeds to emulate the shot noise of the electron beam. In all cases the XFEL process reaches saturation within the available undulator length. As an example, Figs. 4 ▸ and 5 ▸ show the XFEL spectrum and power profile, respectively, at the end of the undulator beamline for one simulation. The total radiation bandwidth is confirmed to be 10%, the total photon pulse duration is 67 fs (*i.e.* the whole electron bunch is lasing), the XFEL peak power is 8.7 ± 1.9 GW, and the pulse energy at the end of the undulator beamline is 146 ± 9 µJ, where the error bar indicates the standard deviation of the results over the simulated seeds.

**Figure 6:**
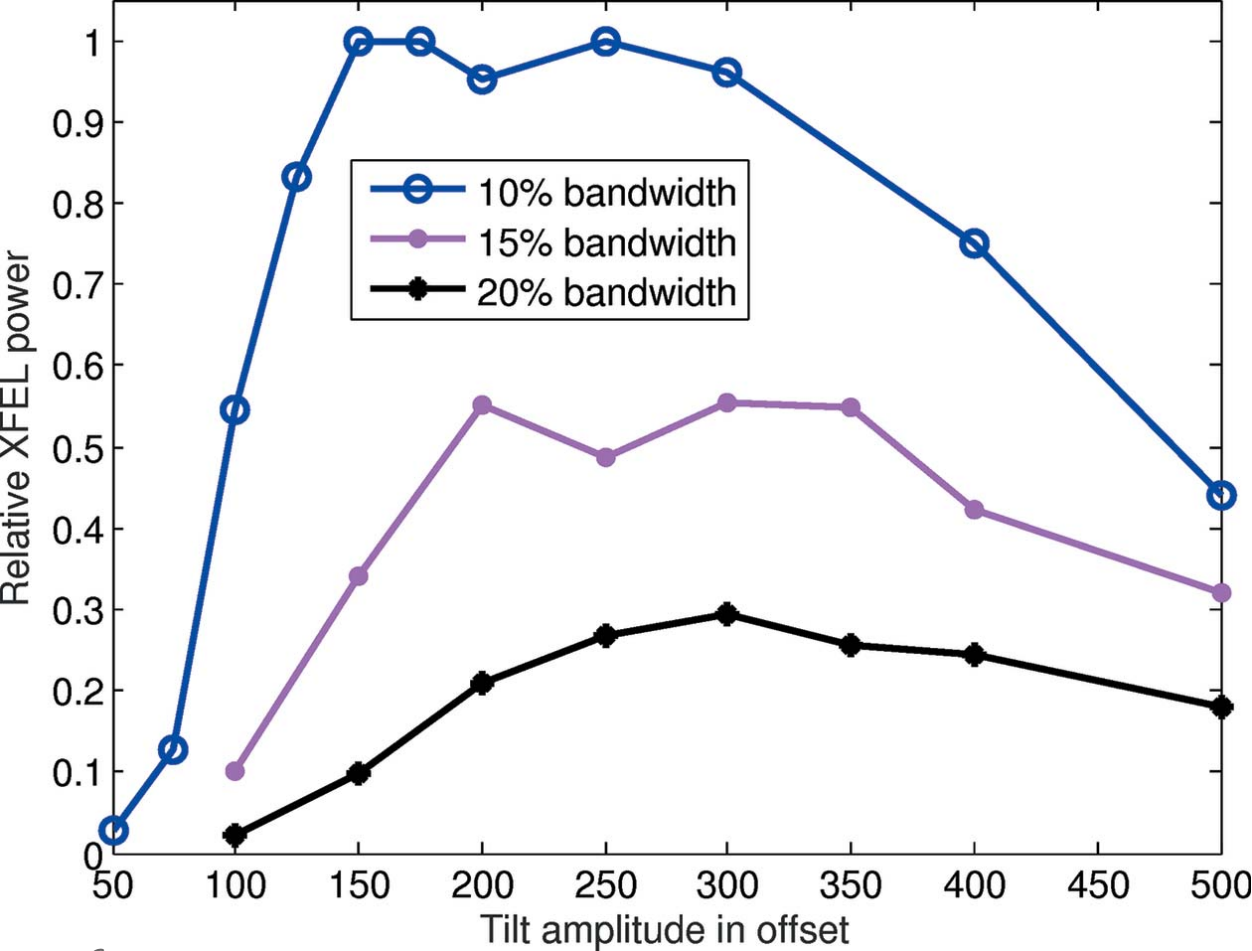
Relative XFEL power as a function of the tilt amplitude to achieve different spectral bandwidths. A relative XFEL power of 1 corresponds to an XFEL pulse energy of 175 µJ. See text for more details.

Obviously a given target XFEL bandwidth can be achieved with different values of the TGU gradient and the beam tilt amplitude, but extreme values in either parameter should be avoided. For instance, in the case of a strong tilt the radiation can only slip a small distance before it escapes the electron beam in the forward direction. If this distance, which scales inversely with the tilt amplitude, is shorter than the cooperation length (Bonifacio *et al.*, 1984 ▸), the XFEL performance is reduced. Similarly, if the gradient is too large, the wavelength change within the intrinsic transverse beam size of the electrons will also be too large, again harming the XFEL performance. We have run numerical simulations to identify the optimal configurations for achieving XFEL pulses with 10%, 15% and 20% bandwidth. Fig. 6 ▸ shows the relative XFEL power as a function of the tilt amplitude in offset for the different bandwidths. For 10% bandwidth, the optimum performance is found with a tilt amplitude in offset between 150 and 300, corresponding to a gradient between 40 m^−1^ and 20 m^−1^. In our previously simulated case with a tilt amplitude in offset of 125, the XFEL power is about 20% below the optimum. As expected, for larger bandwidths (15% and 20%) the achievable XFEL power decreases. The maximum bandwidth that can be realised with our method depends on the minimum power that is required. In our case, we could achieve XFEL pulses with 20% bandwidth and peak powers at the few-gigawatt level. In general, the optimum gradient/tilt amplitude values will depend on various other parameters, such as, for instance, the central radiation wavelength: for a longer wavelength the cooperation length is larger, thereby reducing the maximum acceptable beam tilt.

## 4. Additional possibilities of the scheme   

In our method, short XFEL pulses with different colors can be obtained by simply adding a slotted foil (Emma *et al.*, 2004 ▸) in front of the undulator beamline. The foil spoils the transverse beam emittance of the electrons in such a way that only the electrons traveling through the slots can generate XFEL radiation. Therefore, by means of a multiple-slotted foil, multiple short XFEL pulses with different radiation wavelengths can be generated. The pulse duration and the time separation of the XFEL pulses can be controlled by modifying the slot length and separation, respectively, and the wavelength difference between the pulses can be varied with the amplitude of the tilt and the TGU gradient. Multiple-color operation is in high demand by the scientific user community: for instance, the generation of two-color XFEL pulses with a controlled short time delay between them can be used for two-color pump–probe experiments, which play crucial roles in research fields such as materials science (Baradaran *et al.*, 2012 ▸), molecular physics and photochemistry (Ullrich *et al.*, 2012 ▸).

The ultra-large-bandwidth XFEL pulses obtained from our method can be compressed using pulse-compression techniques. Such techniques, available for some time in the optical regime, are presently being investigated in view of their efficiency for X-rays (see, for instance, Chapman & Nugent, 2002 ▸; Hrdý & Oberta, 2013 ▸). Considering that the intrinsic bandwidth of the XFEL pulses is of the order of 10^−3^ (soft X-rays) and 10^−4^ (hard X-rays) and that the pulses obtained from our method have a total bandwidth of 10% or more, the pulse length could potentially be compressed by a factor of ∼100 (soft X-rays) and ∼1000 (hard X-rays). Consequently, our scheme could in principle be used to obtain multi-terawatt-attosecond XFEL pulses.

## 5. Conclusion   

In summary, we have presented a new and simple method that is able to generate XFEL pulses with ultra-large-bandwidth above the 10% level. The large bandwidth is achieved by sending a transversely tilted beam through a TGU. In this scheme the bandwidth can easily be tuned by modifying the tilt amplitude and/or the gradient of the TGU. Our numerical simulations demonstrate the validity of the method for the SwissFEL case. The novel technique opens up new opportunities for experiments requiring broad-bandwidth XFEL radiation or XFEL pulse trains with multiple colors.

## Figures and Tables

**Figure 5:**
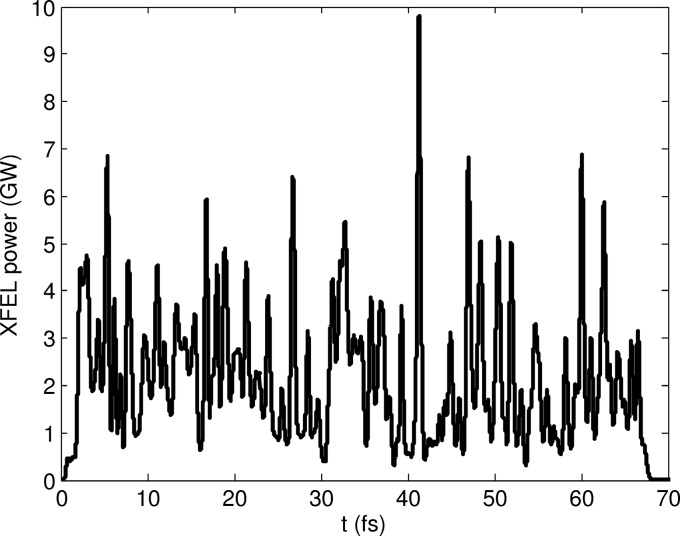
Power profile corresponding to the spectrum shown in Fig. 4 ▸.
